# In Vitro Profiling of Toxicity Effects of Different Environmental Factors on Skin Cells

**DOI:** 10.3390/toxics12020108

**Published:** 2024-01-27

**Authors:** Minghui Fu, Yingxin Yang, Xiaolan Zhang, Bingli Lei, Tian Chen, Yuanqi Chen

**Affiliations:** 1 Institute of Environmental Pollution and Health, School of Environmental and Chemical Engineering, Shanghai University, Shanghai 200444, China; fmh2817@163.com (M.F.); yangyingxin2021@163.com (Y.Y.); zhangxiaolan@shu.edu.cn (X.Z.); 2 State Environmental Protection Key Laboratory of the Assessment of Effects of Emerging Pollutants on Environmental and Human Health, Shanghai Municipal Center for Disease Control and Prevention, Shanghai 200336, China; 3 Department of Environmental Health, Shanghai Municipal Center for Disease Control and Prevention, Shanghai 200336, China; 4 Skincare Research Center of Dr. YU, Shanghai Jahwa United Co., Ltd., Shanghai 200082, China; chenyuanqi@jahwa.com

**Keywords:** FB cells, HaCaT cells, 3D epidermal cells, environmental factors, cytotoxicity

## Abstract

The skin is constantly exposed to a variety of environmental threats. Therefore, the influence of environmental factors on skin damage has always been a matter of concern. This study aimed to investigate the cytotoxic effects of different environmental factors, including cooking oil fumes (COFs), haze (PM_2.5_), and cigarette smoke (CS), on epidermal HaCaT cells and dermal fibroblast (FB) cells. Cell viability, intracellular reactive oxygen species (ROS) generation, inflammatory cytokine levels, and collagen mRNA expression were used as toxicity endpoints. Additionally, the effects of ozone (O_3_) on cell viability and release of inflammatory cytokines in 3D epidermal cells were also examined. The results showed that the organic extracts of CS, COFs, and PM_2.5_ significantly inhibited the viability of HaCaT and FB cells at higher exposure concentrations. These extracts also increased intracellular ROS levels in FB cells. Furthermore, they significantly promoted the release of inflammatory cytokines, such as IL-1α and TNF-α, in HaCaT cells and down-regulated the mRNA expression of collagen I, III, IV, and VII in FB cells. Comparatively, SC organic extracts exhibited stronger cytotoxicity to skin cells compared to PM_2.5_ and COFs. Additionally, O_3_ at all test concentrations significantly inhibited the viability of 3D epidermal cells in a concentration-dependent manner and markedly increased the levels of TNF-α and IL-1α in 3D epidermal cells. These findings emphasize the potential cytotoxicity of COFs, PM_2.5_, CS, and O_3_ to skin cells, which may lead to skin damage; therefore, we should pay attention to these environmental factors and take appropriate measures to protect the skin from their harmful effects.

## 1. Introduction

The skin, consisting of the epidermis and dermis, is the outermost organ of the body. The outer epidermis contains keratinocytes, while the inner dermis is primarily composed of fibroblasts and connective tissues [1]. Serving as a barrier against physical and environmental factors, the skin is constantly exposed to harmful environmental stressors, which can lead to accelerated premature intrinsic skin aging and even skin carcinogenesis [2,3,4].

Among environmental factors, fine particulate matter (PM_2.5_) in haze is a mixture of many harmful ingredients. Some of these harmful ingredients can act as catalysts for the production of reactive oxygen species (ROS) and promote skin inflammation [4,5]. Cigarette smoke (CS) aerosol is formed during smoking due to incomplete combustion [6]. The complex CS aerosol contains over 7000 harmful substances and is classified as a Group A carcinogen by the USEPA [7]. The oxidative compounds in CS can disrupt cellular redox homeostasis, further affect the cutaneous tissue, and have a clear relationship with premature skin aging [8,9]. Oil fumes from high-temperature cooking can form aerosols through the vapor-to-particle process in a temperature gradient region [10]. Exposure to cooking oil fumes (COFs) may have carcinogenic effects on the respiratory system in humans. Epidemiological studies suggest that carcinogens produced during cooking may be one of the factors causing lung cancer in Chinese women [11,12]. Ozone (O_3_) is one of the most reactive environmental oxidants that can come into contact with skin [13]. Previous studies have shown that exposure to O_3_ can result in the depletion of antioxidants and the oxidation of lipids and proteins in the outermost layer of skin, the stratum corneum [14,15].

More than 90% of the urban population is exposed to contaminant concentrations above the standard limits established by the World Health Organization (WHO) [16]. In recent years, the adverse effects of environmental factors such as PM_2.5_, CS, COFs, and O_3_ on the respiratory system in animals and humans have attracted the attention of many researchers [17,18,19,20]. Particularly, the impact of environmental factors on the spread of COVID-19 has become a hot topic in the past two years [21,22,23]. Additionally, several studies have been conducted on the biological effects of some environmental factors such as PM, CS, and O_3_ on skin cells. However, there is a lack of research on the effects of COFs on the epidermis and dermis of the skin. Furthermore, there is a lack of comparative studies on the cytotoxicity of different environmental factors on skin cells.

The epidermal HaCaT cells and dermal fibroblasts (FB cells) are ideal cell models for studying epidermal and dermal skin damage caused by pollutants. Fibroblasts, as one of the most important types of cells in the skin, not only synthesize and secrete collagen to provide physical support for the skin but also participate in tissue repair when the skin is damaged [24]. Increased ROS generation and decreased activity and collagen secretion of fibroblasts have been identified as important factors in wrinkle formation [24]. HaCaT cells play a role in maintaining skin barrier homeostasis [25]. Under the stimulation of environmental factors, HaCaT cells can produce a large number of cytokines, including TNF-α, IL-1α, and IL-6, which can damage the skin tissue [26]. Therefore, in this study, dermal fibroblasts (FB cells) were used to evaluate the effects of PM_2.5_, COFs, and CS on ROS generation and mRNA levels of collagen genes, while HaCaT cells were used to evaluate the effects of PM_2.5_, COFs, and CS on the secretion of cytokines (TNF-α and IL-1α). Additionally, 3D epidermal cells were employed to investigate the influence of O_3_ on skin tissue damage. The findings of this study will highlight the hazards of different environmental factors on human skin.

## 2. Methods and Materials

### 2.1. Reagents

2′,7′-dichlorodihydrofluorescein diacetate (DCFH-DA), ter-butyl hydroperoxide (t-BHP), *tert*-butyl hydroperoxide (t-BHP), and 3-(4,5)-dimethylthiazol (-2-y1)-2,5-diphenyltetrazolium bromide (MTT) were obtained from Sigma (Saint Louis, MO, USA). The SYBR green dye and reverse-transcription kit were purchased from TOYOBO (Osaka, Japan). The ELISA kits for IL-1α and TNF-α were purchased from Mibio Co. (Shanghai, China). All the other reagents used in this study were of analytical grade, unless otherwise specified. The 3D epidermal model used in this study was obtained from Shanghai Jahwa United Co., Ltd. and is a three-dimensional cellular system of human epidermal keratinocytes (HEK).

### 2.2. Sample Collection and Preparation

Fine particulate matter (PM_2.5_) samples were collected using a Th-150c Ⅲ medium-flow atmospheric sampler with a quartz filter membrane for 24 h. The PM_2.5_ samples were collected on 14 December 2019, in Shanghai, China, with an air concentration of 57 μg/m^3^. For cigarette smoke (CS) collection, smoke smog was generated in a closed room by recruited volunteers who smoke and the smog was collected using an atmospheric sampler with a quartz filter membrane. Cooking oil fumes (COFs) were also collected during cooking with an atmospheric sampler with a quartz filter membrane. After collection, the samples were immediately transferred to the laboratory and stored at −20 °C until further preparation.

The PM_2.5_, CS, and COFs adsorbed on the quartz filter membrane were freeze-dried. The quartz filter membranes containing these samples were then cut into pieces and extracted for 24 h using n-hexane/dichloromethane (1/1, *v*/*v*) by the microwave ultrasound method. The organic extracts were reduced to approximately 1 mL using a rotary evaporator (R-200 rotary evaporator, Büchi Labortechnik AG, Flawil, Switzerland) and further concentrated to approximately 10 μL using a gentle nitrogen flow. The solvent in the extracts was then exchanged with 200 μL dimethyl sulfoxide (DMSO). The organic extracts were stored at −20 °C until the toxicity test. Four concentration sequences were prepared by two-fold dilution from stock solutions for the toxicity test. Therefore, the concentration sequences for the PM_2.5_ were 6.375, 12.75, 25.5, and 51 μg/mL, for the CS were 3, 6, 12, and 24 μg/mL, and for the COFs were 101.25, 205.5, 405, and 810 μg/mL. The final DMSO concentration used in the toxicity test was 0.1%, which was non-toxic to FB cells and HaCaT cells.

### 2.3. Maintenance of Skin Cells and MTT Assay

MTT is a commonly used method to detect the cell proliferation and cytotoxicity of chemicals due to its high sensitivity and ease of use. In this study, FB and HaCaT skin cells were obtained from Shanghai Jahwa United Co., Ltd. (Shanghai, China). FB and HaCaT cells were maintained in high glucose Dulbecco’s modified Eagle’s medium (DMEM) (Gibco, Grand Island, NY, USA) with 10% fetal bovine serum (FBS) (Gibco, Grand Island, NY, USA) and 100 units/mL penicillin/streptomycin (Invitrogen, Carlsbad, CA, USA). The cells were incubated in a 5% CO_2_ incubator at 37 °C and the culture medium of the cells was refreshed every 2–3 days. The cells in their exponential growth phase were employed for the toxicity test of organic extracts. A total of 0.1% DMSO was used as the control group.

To perform the MTT assay, HaCaT and FB cells were seeded in 96-well plates with 200 μL culture medium at a density of 3 × 10^3^ cells per well and allowed to adhere for 18–24 h. Subsequently, the cells were treated with different concentrations of organic extracts obtained from the PM_2.5_, CS, and COFs for 24 h. The experiment included a control group treated with 0.1% DMSO and exposure groups with the environmental factors. Each group had six parallel samples. After exposure, the MTT assay was conducted according to the specific experimental process and the calculation method described in our previous study [27].

### 2.4. Reactive Oxygen Species (ROS) Detection

DCFH-DA is a fluorescent probe that has the ability to penetrate cell membrane and enter the cells. Once inside the cells, it is enzymatically hydrolyzed into DCFH (2′,7′-dichlorodihydrofluorescein). In the presence of reactive oxygen species (ROS), DCFH is oxidized and converted into highly fluorescent 2′,7′-dichlorofluorescein (DCF). Therefore, the average fluorescence intensity of cells can be used as an indicator of the instantaneous content of ROS within the cells.

To detect intracellular ROS levels, FB cells in the logarithmic growth stage were seeded in 6-well plates with a volume of 1 mL at a density of 1.5 × 10^5^ cells per well. The cells were cultured overnight in a 5% CO_2_ incubator at 37 °C to allow them to adhere. After adherence, the culture medium was discarded and fresh medium containing different concentrations of organic extracts obtained from the PM_2.5_, CS, and COFs was added to treat the cells for 24 h. The specific experimental method of intracellular ROS detection can be found in our previous study [28]. Briefly, the treated cells were incubated with DCFH-DA and average fluorescence intensity was calculated using Image-pro Plus 6 software based on the fluorescence images obtained using a fluorescence microscope (Olympus BX-51; Olympus, Tokyo, Japan). Each treatment, DMSO negative control, or t-BHP positive control, consisted of three parallel samples.

### 2.5. Determination of TNF-α and IL-1α Content by Sandwich ELISA

ELISA kits were employed to detect the IL-1α and TNF-α levels in the supernatant of the HaCaT cells. The HaCaT cells were seeded in 96-well plates at a density of 1 × 10^5^ cells per well overnight. After adhesion, the cells were treated with different concentrations of the organic extracts obtained from the PM_2.5_, CS, and COFs for 24 h. After treatment, conditioned media from the cell cultures were collected by centrifugation. The ELISA kits were prepared by allowing them to equilibrate for 30 min at room temperature. Then, 100 μL of standard solution and supernatant from each exposure group of environmental factors were added into the standard well and sample well, respectively, which were coated with the primary antibody. The plates were incubated for 2 h at 37 °C. After incubation, the cells were washed with D-Hanks solution and 100 μL of biotin-labeled antibody working solution was added to each well. The plates were then incubated for 1 h at 37 °C. Subsequently, the liquid in each well was discarded and 100 μL of horseradish peroxidase labeled avidin working solution was added to each well and incubated for 1 h at 37 °C. After incubation, 90 μL of substrate solution was added to each well and incubated for 10–30 min at 37 °C in the dark. Finally, 50 μL of termination solution was added to stop the reaction. The optical density (OD) values at a wavelength of 450 nm were measured and the TNF-α and IL-1α levels were calculated based on the OD values of the exposure group and the control group.

### 2.6. RT-qPCR

FB cells in the logarithmic growth stage were seeded at the density of 1 × 10^6^ cells/well and exposed to different concentrations of the organic extracts obtained from the PM_2.5_, CS, and COFs for 12 h. After exposure, RNA samples were collected according to the method described in our previous study [27]. The A_260_/A_280_ ratios for all samples ranged from 1.78 to 2.25, indicating that the extracted RNA is suitable for further reverse-transcriptase polymerase chain reaction (RT-PCR).

The isolated RNA was used to perform RT-qPCR to obtain cDNA according to the instructions provided in the reverse-transcription kit manual. The cDNA was then used for second-strand synthesis and subsequent amplifications.

The PCR amplification was performed using the ABI 7500 fast system. The housekeeping gene β-actin was used as the internal reference gene for subsequent gene expression analyses. The relative expression of the target genes was calculated according to the equation: R = 2^−ΔΔCt^ [29]. Specific primers for target genes, including collagen I (forward: GGACACAGAGGTTTCAGTGG; reverse: CCAGTAGCACCATCATTTCC), III (forward: TTGAAGGAGGATGTTCCCATCT; reverse: ACAGACACATATTTGGCATGGTT), IV (forward: GGGATGCTGTTGAAAGGTGAA; reverse: GGTGGTCCGGTAAATCCTGG), and VII (forward: CAGCGACGTTCTACGGATCA; reverse: TGGGAGTATCTGGTGCCTCA) were obtained from Wcgene Biotechnology Co., Ltd. (Shanghai, China).

### 2.7. Cell Viability and IL-1α and TNF-α Levels in 3D Epidermal Cells

The 3D epidermal cells were exposed to O_3_ in a closed glove box. The O_3_ was generated using O_2_ through an ozone generator. The O_2_ and O_3_ mixtures, consisting of approximately 95% O_2_ and 5% O_3_, were continuously supplied to a Teflon-lined glove box at a flow rate of 70 L/min. The concentrations of O_3_ in the glove box were adjusted to 500, 5000, and 50,000 mg/m^3^, respectively. At the same time, 5% SDS medium and 5% PBS medium were added to separate 6-well plates as positive control and negative control, respectively. Each group, including the control groups and the O_3_-exosed groups, consisted of three parallel samples.

Before O_3_ exposure, the 3D epidermal cell model was washed with PBS and the residual PBS was wiped off inside and outside the model. Then, the 3D epidermal cell model was transferred to a new 6-well plate and 0.9 mL of culture solution was added to each well. The 6-well plate, containing the 3D epidermal cell model, was then exposed to different concentrations of O_3_ (500, 5000, and 50,000 mg/m^3^) for 1 h.

After exposure, the 3D epidermal cell model was transferred to another new 6-well plate. Then, 0.9 mL of culture solution was added to each well, and the plate was placed in an incubator for 24 h. After incubation, the supernatant was collected for the detection of IL-1α and TNF-α levels using ELISA kits. The method for detecting IL-1α and TNF-α levels is described in the previous Section (TNF-α and IL-1α levels).

The 3D epidermal cell model was transferred to a new 24-well plate to further detect cell viability. A total of 300 μL of MTT (1 mg/mL) was added and incubated for 3 h. After incubation, the MTT solution was removed from the 24-well plate, and the 3D epidermal cell model was washed three times with PBS. The surface of the model was carefully dried and the cell model was then transferred to another new 24-well plate. Next, 2 mL of isopropanol was added to each well containing the 3D epidermal cell model and the plate was covered with a sealing film to prevent evaporation. The plate was shaken at room temperature for 2 h at a speed of 120 rpm. Then, the cell model was pierced and blown three times. Subsequently, 200 μL of the purple formazan derivative was sucked out from the 24-well plate and transferred to a 96-well plate. The absorbance at 570 nm was measured.

### 2.8. Data Analysis

The experimental data were characterized as the mean ± standard deviation (SD). To determine the significance between the treatment group and the control group, a one-way analysis of variance (ANOVA) was performed using SPSS 19.0 (Chicago, IL, USA). A *p*-value of less than 0.05 was considered statistically significant.

## 3. Results

### 3.1. Effect of Different Environmental Factors on Cell Viability

**Figure 1:**
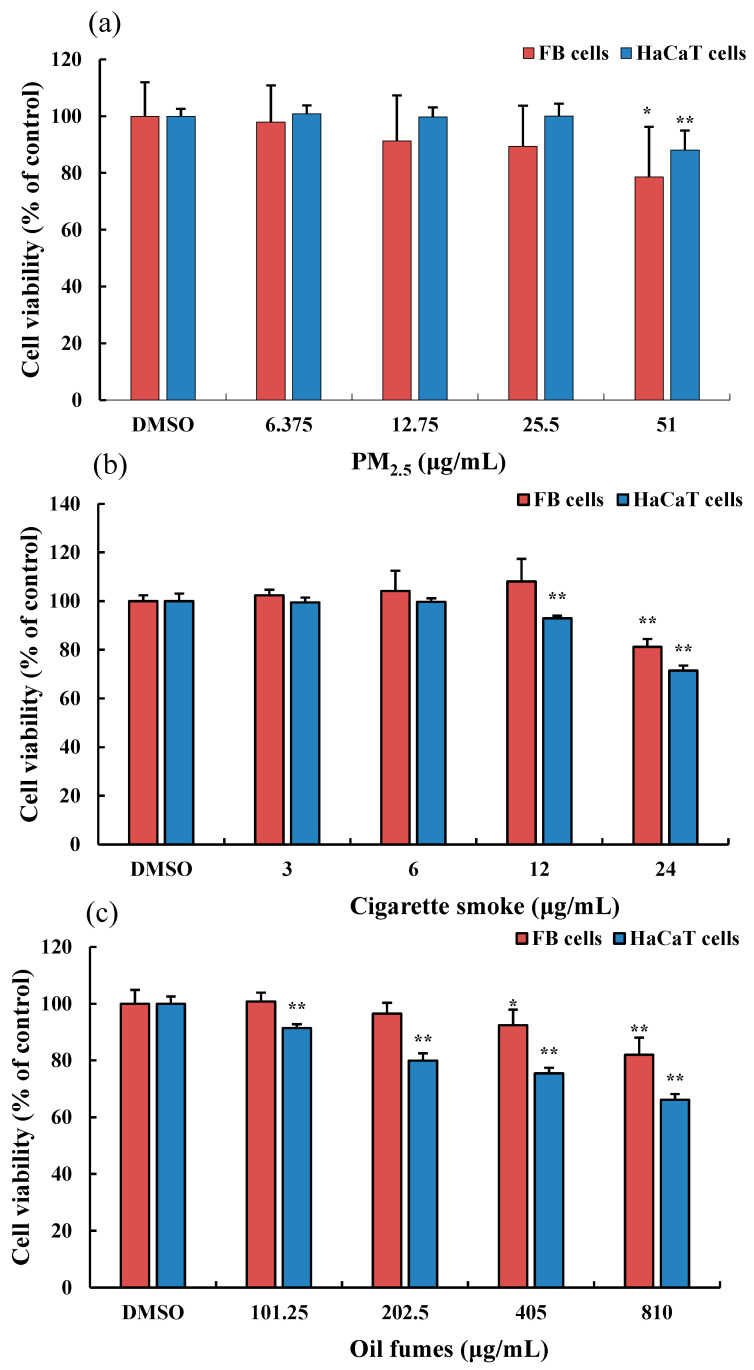
Effect of PM_2.5_ on the viability of FB and HaCaT cells. (**a**): the organic extracts of fine particulate matter (PM_2.5_); (**b**): the organic extracts of cigarette smoke (CS); and (**c**): the organic extracts of cooking oil fumes (COFs). Data are shown as the mean ± standard deviation (SD) of six parallel samples. * *p* < 0.05, ** *p* < 0.01, compared with the negative control (0.1% DMSO).

We evaluated the effect of various environmental factors on cell viability. After incubation for 24 h with different concentrations of PM_2.5_, we found that PM_2.5_ at 6.375, 12.75, and 25.5 μg/mL had no significant cytotoxicity on HaCaT and FB cells, with cell viability maintained above 90%. However, at a concentration of 51 μg/mL, the PM_2.5_ significantly inhibited the viability of HaCaT and FB cells (Figure 1A). When exposed to CS for 24 h at concentrations of 3, 6, and 12 μg/mL, there was no significant cytotoxicity on FB cells. However, at a concentration of 24 μg/mL, the CS significantly inhibited the viability of FB cells. For HaCaT cells, exposure to CS at concentrations of 6 and 24 μg/mL significantly inhibited the HaCaT cell viability (Figure 1B). At a concentration of 101.25 μg/mL, COFs had no significant effect on the viability of FB cells and HaCaT cells. However, at concentrations of 405 and 810 μg/mL, COFs significantly decreased the proliferation of FB cells, and at concentrations of 205.5–810 μg/mL, COFs significantly inhibited the viability of HaCaT cells (Figure 1C). When exposed to 810 μg/mL COFs, FB cell viability decreased by 15% and HaCaT cell viability decreased by 20%. Our results showed that lower concentrations of organic extracts from different environmental factors had no effect on the viability of FB cells and HaCaT cells, while higher concentrations attenuate their survival rates. Among the three environmental factors, CS had the strongest toxicity effects on skin cells compared to PM_2.5_ and COFs, while COFs had the lowest cytotoxicity.

The highest concentrations of CS, PM_2.5_, and COFs in this study significantly inhibited the viability of two skin cells. Therefore, in subsequent intracellular ROS detection in FB cells, the highest concentrations of CS, PM_2.5_, and COFs were set as exposure concentrations.

### 3.2. Effects of Environmental Factors on ROS Levels and the mRNA Expression of Collagen Genes in FB Cells

**Figure 2:**
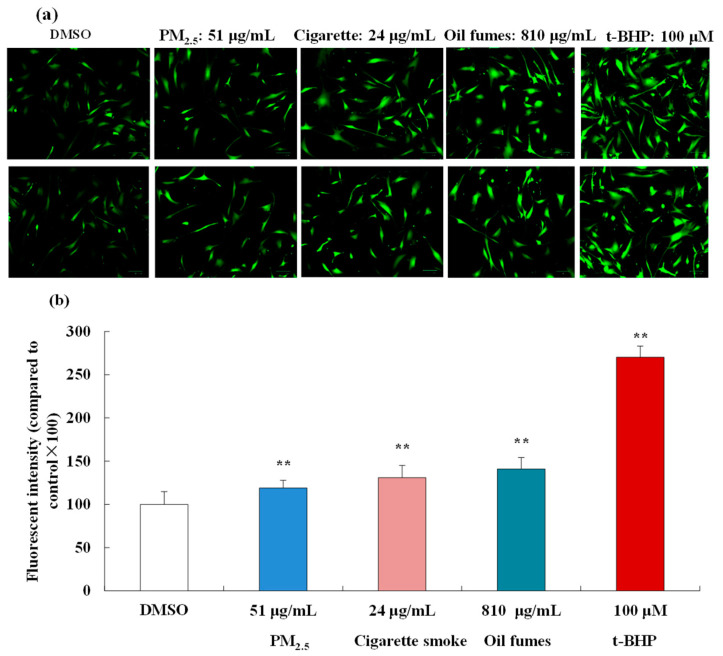
Effect of the PM_2.5_, COFs, and CS on the levels of intracellular ROS in FB cells. (**a**): some fluorescent images of FB cells; and (**b**): ROS levels in FB cells. The scale bar is 20 μm. Data are shown as the mean ± S.D. of three parallel samples. ** *p* < 0.01, compared with negative control (0.1% DMSO).

Based on the cell viability results, 51 μg/mL PM_2.5_, 24 μg/mL CS, and 810 μg/mL COFs were selected as exposure concentrations to further investigate the effect of these three environmental factors on intracellular ROS generation in FB cells. In Figure 2a,b, we observed that t-BHP (100 μM), used as a positive control, significantly elevated intracellular ROS levels. In addition, the PM_2.5_, COFs, and CS at test concentrations were found to significantly induce ROS generation in FB cells. The fluorescence images also clearly showed that various organic extracts promoted intracellular ROS generation. The levels of ROS induced by 24 μg/mL CS were similar to those induced by 54 μg/mL PM_2.5_ and 810 μg/mL COFs, indicating that CS had stronger toxicity compared to the other two environmental factors.

**Figure 3:**
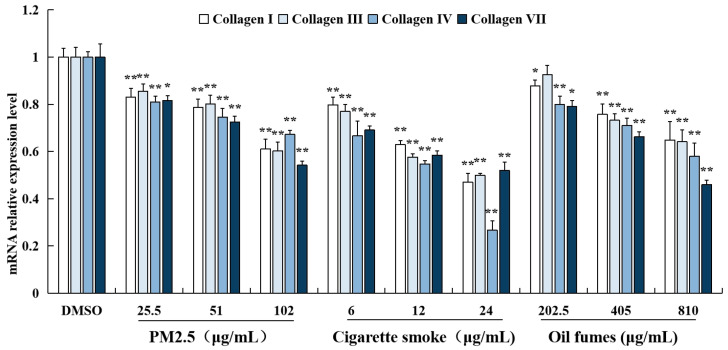
Effect of PM_2.5_, COFs, and CS on the mRNA levels of collagen genes in FB cells. Data are characterized as the mean ± SD of four parallel samples. * *p* < 0.05, ** *p* < 0.01, compared with negative control (0.1% DMSO).

To examine the effects of environmental factors on collagen in FB cells, collagen I, III, IV, and VII were chosen as target genes, as they are associated with skin damage and aging. The mRNA levels of these collagen genes were detected by RT-qPCR technology (Figure 3). Our results showed that exposure to PM_2.5_, CS, and COFs at all test concentrations significantly decreased the mRNA levels of collagen I, III, IV, and VII. The only exception was that COFs at 202.5 μg/mL had no effect on the expression of collagen III mRNA. These findings suggest that exposure to these environmental factors can have a negative impact on the expression of collagen genes in FB cells, which may contribute to skin damage and aging.

### 3.3. Effects of Environmental Factors on the Levels of TNF-α and IL-1α in HaCaT Cells

**Figure 4:**
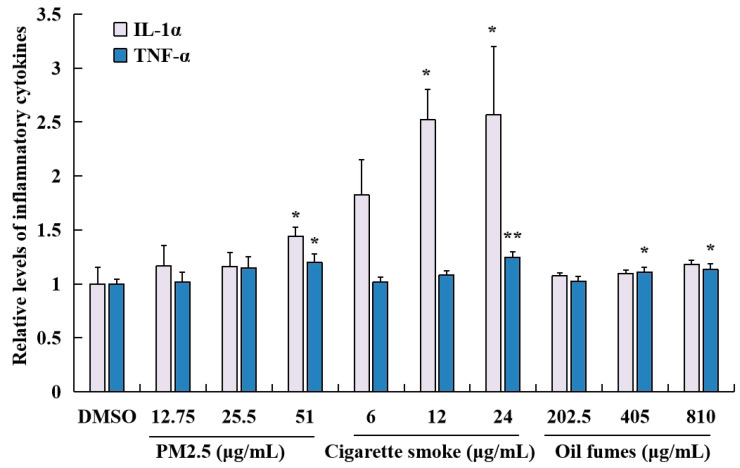
Effect of PM_2.5_, COFs, and CS on the release of IL-1α and TNF-α in HaCaT cells. Data are characterized as the mean ± SD of three parallel samples. * *p* < 0.05, ** *p* < 0.01, compared with negative control (0.1% DMSO).

To evaluate the impact of different environmental factors on the release of pro-inflammatory cytokines, ELISA kits were employed to measure the levels of TNF-α and IL-1α in HaCaT cells. As shown in Figure 4, PM_2.5_ at 51 μg/mL significantly elevated the levels of IL-1α and TNF-α. CS at concentrations of 12 and 24 μg/mL significantly increased the IL-1α levels, while at 24 μg/mL it also increased TNF-α levels. COFs at concentrations of 405 and 810 μg/mL induced significant release of TNF-α, but had no effect on the levels of IL-1α. Furthermore, the levels of IL-1α and TNF-α induced by CS were higher than those induced by PM_2.5_ and COFs. These results indicate that all three environmental factors can significantly promote the release of TNF-α or IL-1α in HaCaT cells, particularly at higher exposure concentrations.

### 3.4. Effect of O_3_ on Cell Viability and IL-1α and TNF-α Release in 3D Epidermal Cells

**Figure 5:**
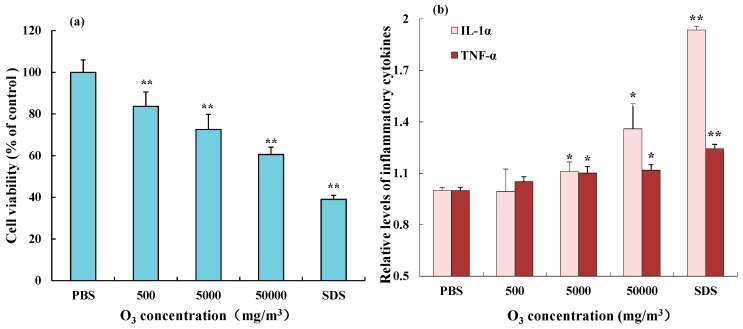
Effect of O_3_ on the cell viability (**a**) and the IL-1α and TNF-α release (**b**) in 3D epidermal cells. Data are characterized as the mean ± SD of three parallel samples. * *p* < 0.05, ** *p* < 0.01, compared with negative control PBS.

Exposure to ozone for 1 h at the test concentrations significantly inhibited the viability of 3D epidermal cells in a concentration-dependent manner (Figure 5a). In addition, ELISA analysis revealed that O_3_ exposure also significantly increased the secretion of IL-1α and TNF-α by 3D epidermal cells in a concentration-dependent manner under the current environmental exposure conditions (Figure 5b).

## 4. Discussion

The skin serves as a crucial barrier that efficiently shields the body from the harmful effects of environmental stressors like air pollutants and ultraviolet rays [30,31]. However, excessive levels of environment stressors can result in skin damage [26,32,33]. Acute exposure to these environmental stressors can trigger the activation of various signaling pathways that coordinate adaptive stress response to maintain the homeostasis of skin cells and tissues [34].

In this study, we found that environmental stressors such as PM_2.5_, COFs, and CS had a dual effect on the viability of FB and HaCaT cells. At low concentrations, these stressors promoted cell viability, while at higher concentrations, they inhibited cell viability. This may be attributed to the toxicity of pollutants present in PM_2.5_, CS, and COFs. PM_2.5_ is a complex mixture of harmful components, including organic pollutants, transition metals, and free radicals. CS is composed of thousands of toxic compounds such as polycyclic aromatic hydrocarbons (PAHs), tobacco-specific nitrosamines, aldehydes (formaldehyde, acrolein, and 4-hydroxynonenal), and various active free radicals [35,36,37]. COFs, which are commonly found in kitchen and indoor air, are considered to be the main contaminants that pose a threat to human health [38]. Therefore, it is reasonable to assume that these environmental factors can inhibit cell growth and induce cytotoxicity.

Environmental pollutants have been shown to induce the excessive production of ROS, leading to various forms of skin damage associated with oxidative stress [39,40]. ROS is directly involved in DNA oxidative damage and lipid peroxidation [41]. In the presence of hydrogen peroxide and light, redox-active metal ions, such as copper and iron, can act as catalysts for the generation of hydroxyl radicals and other ROS. These ROS can disrupt the redox balance of cells and contribute to different skin diseases [42]. In this study, we observed that PM_2.5_, CS, and COFs increased the levels of intracellular ROS in FB cells. This suggests that these environmental factors can disrupt the cellular redox homeostasis, further supporting their potential to induce oxidative stress and contribute to skin damage.

In addition to disrupting the balance of cellular redox, some environmental factors can also evoke inflammation in the skin. For example, Soeur et al. [42] found that components of cigarette smoke can induce the production of pro-inflammatory mediators. They found that numerous chemicals, such as PAHs, can readily penetrate the outer layer of the skin and enter the bloodstream through the capillaries in the dermis. Once in the blood stream, they can stimulate the production of pro-inflammatory mediators, leading to adverse effects on the skin tissue. Chronic inflammatory reactions are then associated with skin disorders like atopic dermatitis and psoriasis [43]. In these conditions, the immune system is activated and releases cytokines that affect the growth and differentiation of skin cells. Several studies have shown that prolonged exposure to O_3_ can increase oxidative damage and promote release of pro-inflammatory cytokines, such as interleukin-1β (IL-1β) and IL-18, leading to various inflammatory skin diseases [44,45]. Our study also observed that PM_2.5_, CS, and COFs inhibited the viability of FB cells and HaCaT cells and increased the release of pro-inflammatory cytokines like TNF-α and IL-1α. In addition, O_3_ inhibited the viability of 3D epidermal cells and elevated the levels of TNF-α and IL-1α in 3D epidermal cells. The findings suggest that PM_2.5_, CS, COFs, and O_3_ may contribute to the development of skin inflammation-related diseases.

The inflammatory changes of HaCaT cells are likely related to the levels of intracellular ROS. In the human skin, numerous contaminants can induce the excessive generation of ROS, which, in turn, causes skin inflammation and various cases of skin damage [46,47,48]. O_3_ is known to produce active free radicals and induce inflammatory responses [49,50]. Ansary et al. [51] found that ultraviolet radiation (UVR) has also been found to cause skin inflammation through ROS. Abais et al. [41] found that exposure to air pollution exacerbates inflammatory skin diseases and ROS seems to play a key role in regulating the inflammasome activity. Environmental factors, such as PM_2.5_, CS, and COFs, include a variety of pollutants. Therefore, it is reasonable to assume that environmental factors can induce inflammatory through increased ROS generation. Mokrzyński et al. [4] found that PM_2.5_ can elevate ROS levels, leading to oxidative stress, inflammation, skin aging, and even skin cancer development. In addition, some studies found that ROS has been shown to induce the release of pro-inflammatory cytokines [10,16], which are important factors in skin inflammation. Therefore, excessive ROS production is considered an important initial step in cell damage, photo-aging, immune system change, and skin carcinogenesis [32].

The main indicator of skin damage is the depletion of collagen. The generation of ROS can affect the synthesis and secretion of collagen. Treatment of human dermal fibroblasts with hydrogen peroxide solution (H_2_O_2_) increases intracellular ROS levels, leading to the expression of matrix metalloproteinase-1 (MMP-1) and a decrease in collagen secretion [52]. Similarly, in this study, PM_2.5_, CS, and COFs were found to down-regulate the mRNA expression of collagen genes and increase intracellular ROS levels in FB cells. This suggests that PM_2.5_, CS, and COFs can decrease collagen secretion by increasing intracellular ROS generation, potentially leading to further skin damage. Kruk and Duchnik [53] found that oxidative stress has been shown to induce chronic inflammatory reaction, which, in turn, can lead to the collagen rupture and dysfunction of collagen fibers and skin cells. Exposure to COFs has been found to significantly increase oxidative stress biomarkers (ROS and MDA), pro-inflammatory markers (TNF-α and IL-1ß), and markers of apoptosis (NF-kappa B and Caspase-3) in rat lungs. The toxicity of COFs on the lungs can be reduced by vitamin E, suggesting that oxidative stress may be primarily responsible for the observed toxicity induced by COFs [20].

These results suggest that ROS may play an important role in the cell viability, release of pro-inflammatory cytokines, and mRNA expression of collagen I, III, IV, and VII induced by PM_2.5_ and COFs. However, in comparison, CS had lower effect on ROS production, but exhibited a major effect on cytokines expression. This indicates that for CS, it is unclear whether ROS play a key role, at least in cytokines expression. Therefore, further investigation is needed to elucidate the mechanism behind these findings. For example, the use of ROS scavengers can help determine the role of ROS in these biological effects by investigating the relationship between cell viability, levels of IL-1α and TNF-α, or the mRNA expression of collagen and ROS generation.

## 5. Conclusions

The main biological effects of acute stressors on skin include alterations in the skin barrier, subclinical microinflammation, inflammation, immunosuppression, DNA damage, melanogenesis, and changes in sebum and sweat production. Environmental factors such as PM_2.5_, CS, and COFs at the test concentrations were found to decrease the viability of FB cells and HaCaT cells. They also promoted the secretion of pro-inflammatory cytokines IL-1α and TNF-α in HaCaT cells, increased intracellular ROS levels, and down-regulated the mRNA expression of collagen I, III, IV, and VII in FB cells. In comparison, among PM_2.5_, CS, and COFs, the organic extract of CS exhibited the strongest cytotoxicity, followed by PM_2.5_. In addition, O_3_ exposure also reduced the viability of 3D skin cells and elevated the levels of IL-1α and TNF-α. These findings are of value as they suggest that these common environmental factors can potentially damage our skin. Therefore, it is important to minimize exposure to these environmental factors to reduce skin damage. These results also offer a new perspective for the development of cosmetics. For instance, cosmetics could be designed to repair skin damage caused by these environmental factors or to provide a protective barrier against exposure to these factors.

## Data Availability

The datasets used and/or analyzed during the current study are available from the corresponding authors on reasonable request.
